# Pilot study of a robotic protocol to treat shoulder subluxation in patients with chronic stroke

**DOI:** 10.1186/1743-0003-10-88

**Published:** 2013-08-05

**Authors:** Carolin I Dohle, Avrielle Rykman, Johanna Chang, Bruce T Volpe

**Affiliations:** 1 The Burke Rehabilitation Hospital, White Plains, NY, USA; 2 Weill Cornell Medical College, New York, NY, USA; 3 Robotics Research Laboratory, The Feinstein Institute for Medical Research, Manhasset, NY, USA

**Keywords:** Stroke, Subluxation, Spasticity, Shoulder, Rehabilitation, Robotics

## Abstract

**Background:**

Shoulder subluxation is a frequent complication of motor impairment after stroke, leading to soft tissue damage, stretching of the joint capsule, rotator cuff injury, and in some cases pain, thus limiting use of the affected extremity beyond weakness. In this pilot study, we determined whether robotic treatment of chronic shoulder subluxation can lead to functional improvement and whether any improvement was robust.

**Methods:**

18 patients with chronic stroke (3.9 ± 2.9 years from acute stroke), completed 6 weeks of robotic training using the linear shoulder robot. Training was performed 3 times per week on alternate days. Each session consisted of 3 sets of 320 repetitions of the affected arm, and the robotic protocol alternated between training vertical arm movements, shoulder flexion and extension, in an anti-gravity plane, and training horizontal arm movements, scapular protraction and retraction, in a gravity eliminated plane.

**Results:**

Training with the linear robot improved shoulder stability, motor power, and resulted in improved functional outcomes that were robust 3 months after training.

**Conclusion:**

In this uncontrolled pilot study, the robotic protocol effectively treated shoulder subluxation in chronic stroke patients. Treatment of subluxation can lead to improved functional use of the affected arm, likely by increasing motor power in the trained muscles.

## Introduction

Glenohumeral subluxation (GHS) occurs commonly in 17- 81% of those with a paralyzed or plegic upper limb after stroke [1-5], in part because the shoulder is stabilized only by surrounding muscles, the joint capsules and ligamentous structures. Typically, the subluxation that occurs after stroke, particularly during the early flaccid phase, is in the inferior direction. This is likely due to the effects of gravity and the basic structure that allows increased laxity in the inferior capsule to afford adequate joint freedom [2]. Functionally, shoulder subluxation is often associated with soft tissue and capsular damage [6], as well as rotator cuff injury, thus limiting the mobility of the already weakened extremity and interfering with patients’ ability to participate in active rehabilitation [7-9]. Studies have shown a causal link between shoulder subluxation and the development of reflex sympathetic dystrophy [7,10]. Other studies have attempted to link shoulder subluxation to hemiplegic shoulder pain (HSP) [8,11-14], but a clear relationship has not been established [1,15-17].

Standard treatment programs in the acute rehabilitation setting include support of the affected extremity with shoulder slings and wheelchair lap trays [18]. However, these procedures fix the affected limb in one position, placing it at risk for adhesive capsulitis and soft tissue contracture. More active treatment strategies utilize shoulder “kinesio” taping, electrical stimulation of the scapulae and rotator cuff muscles, and shoulder mobilization by positioning the arm. These treatments aim to strengthen the muscles of the rotator cuff and the scapula [19,20] but the effects are transient [1]. Because increased intensity of motor training, especially with robotic devices [21], appears to improve functional outcome after stroke activity, we tested in an uncontrolled pilot trial whether training motor control of the proximal shoulder and scapula with an anti-gravity shoulder robot protocol would alter shoulder subluxation.

## Methods

### Patients and protocol

**Figure 1:**
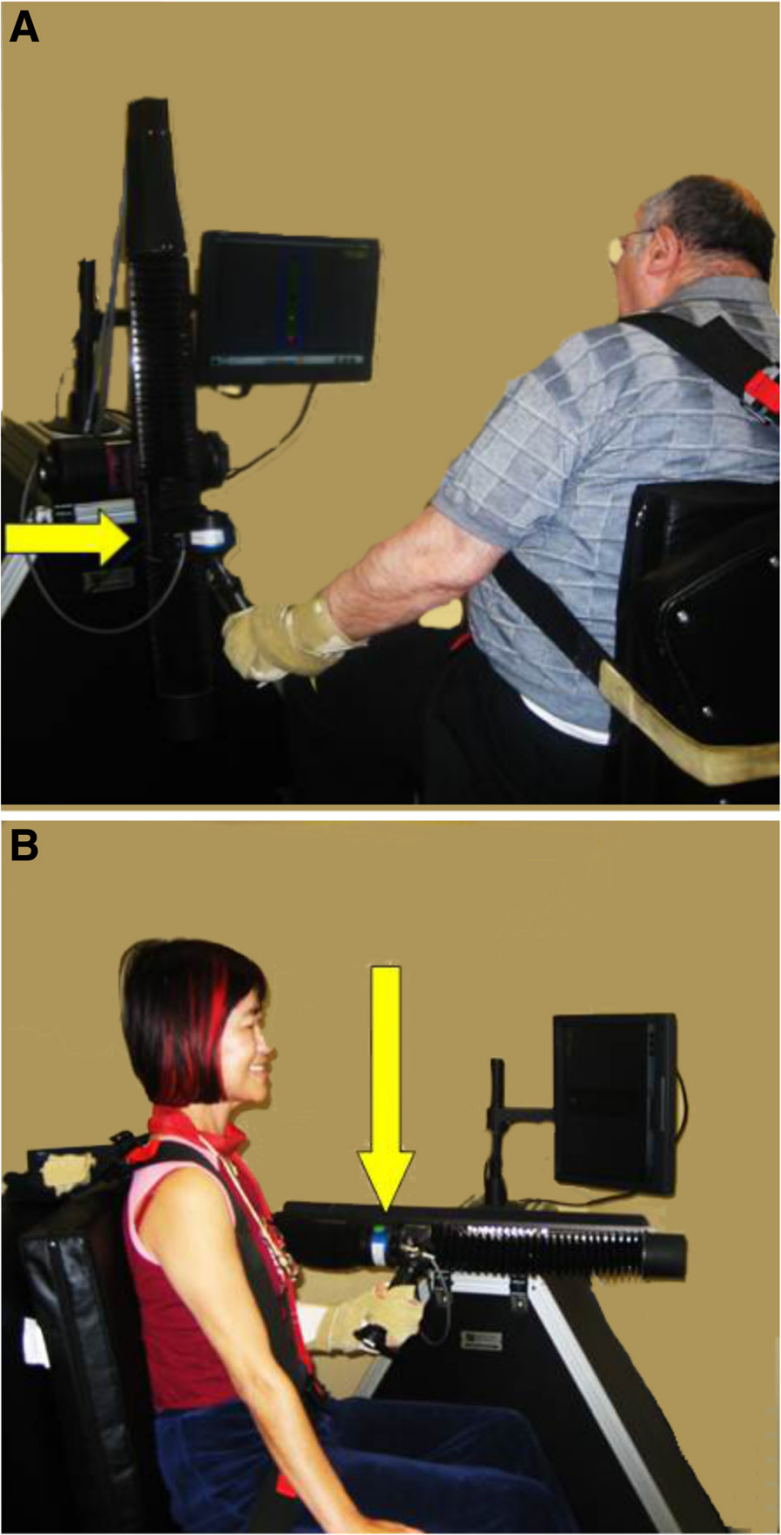
**A**. **A patient working with the linear robot in the vertical plane (arrow locates the vertical sliding plane and the hand-machine contact).** The patient is placed in a comfortable seated position, and compensatory torso movements are minimized by use of a seatbelt as well as constant supervision during the training by a skilled therapist. Visual feed-back is provided on a computer screen by a yellow ball that the patient has to move between targets. **B**. Demonstration of use of the robot in the horizontal, gravity eliminated plane (arrow locates the sliding plane and the point of hand-machine contact).

18 patients, 12 with chronic ischemic and 6 with chronic hemorrhagic stroke (3.9±2.9 years from acute stroke; 63.7±15.9 years; 8 females, 10 males), were included in this study (Table 1). Inclusion criteria were glenohumeral instability in the chronic (>12 months) phase after stroke. Patients with intact glenohumeral joint, inability to follow 1-2 step commands and a fixed contracture in any joint of the affected extremity were excluded. All patients completed 6 weeks of robotic training using the linear shoulder robot (IMT, Cambridge, MA). Patients were seated in a comfortable position either facing the robot or with the robot next to their affected side (Figure 1). Their affected hand was secured to the robot handle in a comfortable flexion grip with the least restriction possible while still guaranteeing stable positioning. A four point seatbelt minimized torso movement. A patient faced the video screen on which a cursor monitored the position of the end of the robot manipulandum to which their hand was fastened. The patient was asked to move their arm along a track to mimic the movement of the cursor on the screen, and the screen provided visual feedback (Figure 1). Each session consisted of 3 sets of 320 repetitions of either vertical arm movements in an anti-gravity plane of movement, or horizontal arm movements in a gravity eliminated plane. The vertical and horizontal training protocolwere alternated across sessions. Training was performed 3 times per week on alternate days. If the patient was unable to complete a movement after 2 seconds, the movement was completed by the robot in an “assisted-as-needed” fashion. All patients included in our study completed the 6 weeks training paradigm. The study was approved by the institutional review board at the Burke Rehabilitation Center and all subjects signed informed consent documents.

**Table 1:** Patient demographics

**Age (mean years ±SD**	**63.7 ± 15.9**
Gender	10 male 8 female
Hemisphere affected	7 Left 11 Right
Years after stroke (mean yrs ±SD)	3.9 ± 2.9
stroke type	12 ischemic 6 hemorrhagic
Ethnicity	15 Caucasian 2 African-Americans 1Hispanic

### Outcome measures

In order to assign a degree of subluxation, we used a standard clinical, non-radiologic method that has high inter-rater reliability [22,23]. Primary outcome measured change in shoulder stability in millimeters (by a single examiner who was not aware of the point of the study) [22,23]. Secondary outcome measures included assessments of affected upper extremity motor function (Fugl Meyer (FM) scale for the upper extremity (UE) divided into shoulder/elbow and wrist/hand cumulative scores [21]). The UE FM score was subdivided into muscles involved in hand/wrist movements vs. shoulder/elbow movements, and changes over time were analyzed separately. Spasticity was recorded using the Modified Ashworth Scale; MAS. The motor power scale was used to evaluate the scapular and rotator cuff muscles. Specifically, an examiner measured scapula abduction/upward rotation (serratus anterior); scapular elevation (upper trapezius and levator scapulae); scapular adduction (middle trapezius and rhomboid major); scapular adduction and depression (lower trapezius); scapular adduction and downward rotation (rhomboid major and minor. Motor power of the rotator cuff was scored in a similar fashion and included shoulder flexion (deltoid, coracobrachialis, rotator cuff), shoulder extension (latissimus dorsi, teres major, posterior deltoid), shoulder abduction (deltoid, supraspinatus), horizontal adduction (pectoralis major), horizontal abduction (infraspinatus, teres minor), and external rotation (infraspinatus, teres minor), and internal rotation (subscapularis, pectoralis major, latissimus dorsi, teres major). Motor power values were summed across the muscle groups. To determine motor power the five scapular muscles were each scored on a 0 (no movement) to 5 (normal strength with full resistance in an anti-gravity position) scale, and were added to form a single score. Outcome measures were obtained on admission to the study, discharge from training, and at a follow up visit 3 months later.

### Data analysis

All data met the Shapiro-Wilk test for normality. Repeated measure ANOVA assessed differences between admission, discharge and follow up exams. Data met Mauchly’s test for sphericity with the exception of the FM scores for wrist/hand, which were corrected (Greenhouse Geiser). If repeated measure ANOVA results were found to be overall significant, Bonferroni post hoc test allowed analysis of which groups were different. All data analysis was performed using program SPSS version 11.5. P values of ≤ 0.05 were considered statistically significant. All results are presented as mean ± Standard Error.

## Results

Training with the anti-gravity shoulder robot decreased subluxation and spasticity, improved functional outcome as captured by significant improvement in the FM scores, and increased motor power. Specifically, assessment of shoulder subluxation showed that subluxation decreased significantly from admission to discharge (56.7±0.3 mm on admission to 26.7±0.2 mm on discharge; p <0.0001 n=18; Table 2A), and persisted at the 3 month follow up visit (33.3±0.3 mm, p=0.001; Table 2A). There was no change between discharge and follow up 3 months later.

**Table 2:** Shoulder stability and mobility outcome after robotic training

**(A) Admission**	**(B) Discharge**	**(C) Followup**	**Confidence Interval**^**a**^	**Significance**^**b**^
A. Measure for shoulder stability (mean in mm ± SEM)				
56.7 (0.3)	26.7 (0.2)	33.3 (0.3)	B-A 1.32-2.68	<0.001
			C-A 0.69-24.2	0.001
B. modified Ashworth Scale (mean ± SEM)				
9 (0.9)	7 (0.9)	8 (1.0)	B-A 0.84 -3.28	0.001
			C-A -4.74-1.77	0.428
C. Fulg- Meyer Assessment, shoulder/elbow (mean ± SEM)				
13.6 (1.2)	15.0 (1.3)	15.0 (1.3)	B-A -2.32- -0.35	0.007
			C-A -2.43- -0.24	0.015
D. Motor power of scapular (mean ± SEM)				
10 (0.9)	12 (1.0)	11 (1.1)	B-A -3.16 - 0.62	0.003
			C-A -2.64 - -0.25	0.016
E. Motor power of rotator cuff muscles (mean ± SEM)				
10 (0.6)	11 (0.8)	11 (0.8)	B-A -2.17 - -0.61	0.001
			C-A -1.84 - -0.05	0.037

**A.** Shoulder stability as measured in mm. (single examiner, blinded to design) demonstrated significant decrease in subluxation between Admission (A) and Discharge (B) evaluations, and between Admission and Follow up (C) evaluations. There was no significant change in the degree of subluxation between Discharge and Follow up exams (3 months later).

**B.** Spasticity assessed with the Modified Ashworth Scale was significantly decreased in between Admission (A) and Discharge (B).

**C.** Upper extremity Fugl Meyer Motor Score was significantly improved between Admission (A) and Discharge (B) evaluations, and between Admission and Follow up (C) evaluations. The increased motor function was stable and robust between discharge and follow up exams (3 months later).

**D**. Motor Power (standard 0-5 scale) for scapular muscles summed the scores over serratus anterior, upper middle and lower trapezius, levator scapulae, major and minor rhomboid, and demonstrated significant improvement from Admission to Discharge that persisted on follow up 3 months later.

**E**. Motor Power (standard 0-5 scale) for rotator cuff muscles summed the scores for deltoid, coracobrachialis,teres major and minor, supraspinatus, infraspinatus, pectoralis major and minor, latissimus dorsi, and demonstrated significant improvement from Admission to Discharge that persisted at followup 3 months later.

^**a**^Adjustment for multiple comparisons: Bonferroni.

^**b**^The mean difference is significant at the 0.05 level.

Spasticity as measured with the combined MAS of the trained shoulder muscles, as well as of the elbow, forearm and wrist, decreased significantly between admission and discharge (9±0.9 admission score to 7±0.9 discharge score; p=0.001 n=17). This effect did not persist at the 3 months follow up (8±1.0, p=0.428 n=17; Table 2B).

FM scores for the shoulder/elbow demonstrated a significant improvement after training (13.6±1.2 admission to 15.0±1.3 discharge; p=0.007, n= 18; Table 2C); an effect that persisted 3 months later (15 ±1.3, p= 0.015, n= 18; Table 2C). There were no significant changes in the FM scores for the wrist/hand (data not shown). Since the anti-gravity robot only trained muscles that are involved in shoulder flexion and extension, scapular protraction and retraction, and the wrist and hand are strapped in a fixed position, this result was not unexpected.

Motor power of scapular and rotator cuff muscles increased significantly from admission to discharge (p=0.003 and p=0.001, respectively; Table 2D and 2E) and persisted at 3 months.

All patients were assessed for the presence of pain at the onset and after completion of the study as well as at follow up. 12 muscle groups were included in the assessment of pain, with pain graded according to the Fugl Meyer Pain Assessment (0=marked pain, 1 = some pain, 2 = no pain). Pain was not a significant problem in our patient population (22.9±2.9, n= 18, with a score of 24 indicating no pain at all in one limb), and thus was not included as an outcome measure in our analysis.

## Conclusion

In this pilot study, the data demonstrate that the treatment protocol with the anti-gravity robot significantly reduced shoulder instability in chronic stroke patients, and the therapeutic effect lasted beyond the duration of treatment. Overall these data suggest that the robotic training strengthened the control of scapular protraction and retraction and shoulder flexion and extension. This emergent stability may underlie the increase in motor power of shoulder muscles. Additionally, this robotic treatment decreased spasticity, which may further increase use of the affected extremity and possibly help prevent complications such as adhesive capsulitis. Improved shoulder stability, decreased spasticity and increased motor power translated into a functional improvement as measured with the upper extremity Fugl Meyer Score for shoulder and elbow function.

Our pilot study did not test whether anti-gravity robot training is superior to conventional physical therapy, although all our patients had completed standard rehabilitation and had persistent significant shoulder subluxation. Benefits of rehabilitation robots in general are that progress can be monitored in real time, since patient’s motor initiation, strength of movement, and accuracy are recorded and can be compared between sessions. Also, rehabilitation robots are engaging and may increase subjects’ motivation to participate in restorative therapy [24]. The questions as to whether the robot provides therapeutic benefit beyond that of conventional physical and occupational therapy will have to be established in future studies.

## Competing interests

None of the authors have any competing interests with this work.398.

## Authors’ contributions

AR and BTV conceived the study; AR, CID, Chang executed the study; CID, AR analyzed the data; BTV, CID, AR, JC wrote the manuscript. All authors read and approved the final manuscript.
